# Supplementary material for: Twin home birth: Outcomes of 100 sets of twins in the care of a single practitioner
Source: PLoS One. 2024 Dec 11;19(12):e0313941. doi: 10.1371/journal.pone.0313941 (PMC11633979; doi:10.1371/journal.pone.0313941)
Supplement: S1 File — This file includes a video link to a twin birth attended by SJF. (DOCX) [file pone.0313941.s002.docx]

**Video of a twin community birth with SJF**

This video is graciously shared by the parents: [https://vimeo.com/160678505](https://vimeo.com/160678505?fbclid=IwAR2f6Rvl0fgZ7TYGEualmKiPD8GlUidjgsKj4tN-x_0DhmfMUYtMKjE4-04)
