# Supplementary material for: Twin home birth: Outcomes of 100 sets of twins in the care of a single practitioner
Source: PLoS One. 2024 Dec 11;19(12):e0313941. doi: 10.1371/journal.pone.0313941 (PMC11633979; doi:10.1371/journal.pone.0313941)
Supplement: S2 File — This file includes details on the six VBAC twin labors. (DOCX) [file pone.0313941.s003.docx]

**Details on VBAC twin labors**

All 6 VBAC twin clients were di-di twins.

**Multip VBACs (1)**

1. G3P2 with breech-breech twins. Uneventful spontaneous vaginal birth of both twins. 5-minute Apgars of 9 & 9 with an intact perineum and 300 ml EBL.

**Functional primip VBACs (5)**

2. G2P1 with vertex-vertex twins. Uneventful spontaneous vaginal birth of both twins, with twin A born in water. 5-minute Apgars of 9 & 9 with a 1^st^ degree laceration and 500 ml EBL.

3. G2P1 with vertex-breech twins. Uneventful spontaneous vaginal birth of both twins. 5-minute Apgars of 9 & 7 with an intact perineum. EBL not available for this birth.

4. G3P2 VBA2C with breech-vertex twins. Uneventful spontaneous vaginal birth of breech twin A and vacuum for twin B due to compound presentation and cord prolapse. 5-minute Apgars of 9 & 10 with a 1^st^ degree laceration and 900 ml EBL.

5. G2P1 with vertex-transverse twins. Transported in early labor due to audible decelerations (transported via car because it was not considered urgent), babies born by C-section.

6. G2P1 with breech-vertex twins. Urgent transfer at full dilation due to sudden onset of constant suprapubic pain. Babies born by C-section; the mother did not have a uterine rupture.
