# Supplementary material for: Twin home birth: Outcomes of 100 sets of twins in the care of a single practitioner
Source: PLoS One. 2024 Dec 11;19(12):e0313941. doi: 10.1371/journal.pone.0313941 (PMC11633979; doi:10.1371/journal.pone.0313941)
Supplement: S3 File — This file describes the circumstances surrounding the eight in-labor transports, of which one was considered emergent. (DOCX) [file pone.0313941.s004.docx]

**History of 8 twin labor transports**

**Nonemergent transport, no option for augmentation, CS (n=4)**

Case #1:

G1P0 IVF Di-Di twins, vertex/vertex. Spontaneous labor at 40 weeks. Nonemergent transport for arrest of labor at 7cm. No option for augmentation at local hospital. Had primary low transverse cesarean section. No complications. Mother and babies discharged routinely.

Case #2:

G2P0 IVF Di-Di twins, vertex/breech. Spontaneous labor at 40 weeks. Nonemergent transport for arrest at <7 cm. No options for augmentation at local hospital. Had primary low transverse cesarean section. No complications. Mother and babies discharged routinely.

Case #3:

G2P1 IVF Di-Di twins, breech/breech. Spontaneous labor at 39 weeks. Arrest at 7cm. No option for augmentation with Br/Br twins. Multip had primary low transverse cesarean section. No complications. Mother and babies discharged routinely.

Case #4:

G3P0 Di-Di twins, vertex/breech. Spontaneous labor at 40 weeks with ROM x 50 hours, arrest at 4cm. Nonemergent transport. No option of augmentation at local hospital. No fever or fetal tachycardia. Had primary low transverse cesarean section. No complications. Mother and babies discharged routinely.

**Nonemergent transport, pVBAC, decels, RCS (n=1)**

Case #5:

G2P1 TOLAC Di-Di twins, vertex/transverse. Spontaneous labor at 42 weeks. Nonemergent transport for audible variable decelerations in early labor. Straight to repeat cesarean section. No evidence of uterine scar separation. No complications. Mother and babies discharged routinely.

**Emergent transport, pVBAC, RCS (n=1)**

Case #6:

G3P1 IVF TOLAC Di-Di twins, breech/vertex. Spontaneous labor at 36 weeks. Sudden onset of sharp suprapubic pain at 10cm. No decelerations or abnormal bleeding. Client opted for transport over concerns about scar dehiscence. Had repeat cesarean section. No complications. No evidence of scar separation. Mother and babies discharged routinely.

**Nonemergent transport for augmentation, vaginal birth (n=2)**

Case #7:

G2P0 IVF donor embryos (maternal age 47) Di-Di twins, vertex/vertex. Spontaneous labor at 41 weeks. Nonemergent transport for stalled labor <4cm. Received augmentation without anesthesia with local MD. Vaginal delivery of twin A, occiput posterior. Vacuum assisted delivery of twin B for decelerations in second stage also occiput posterior (3 hours and 53 minutes apart). Baby B went to NICU for observation. Postpartum hemorrhage, no transfusion. Perineal laceration, repaired. Mother and baby A discharged routinely. Supplemented with donor milk at home. Baby B discharged after 6 days.

Case #8:

G2P0 Di-Di twins, vertex/vertex. Spontaneous labor at 37 weeks. Nonemergent transport after prolonged latent/early labor and exhaustion. Dilated <4cm. Received CE, augmentation, had spontaneous vaginal delivery of both twins 6 hours and 5 minutes apart. Intact. No complications. Mother and babies discharged routinely.
