# Supplementary material for: Twin home birth: Outcomes of 100 sets of twins in the care of a single practitioner
Source: PLoS One. 2024 Dec 11;19(12):e0313941. doi: 10.1371/journal.pone.0313941 (PMC11633979; doi:10.1371/journal.pone.0313941)
Supplement: S4 File — This file describes the two postpartum transports: one neonatal transport for cyanosis and persistent tachypnea and one maternal transport for seizures. (DOCX) [file pone.0313941.s005.docx]

**Details on neonatal & maternal transports**

**Details on newborn TTN transport**

After an uneventful delivery of twin A with a 5-minute Apgar score of 9, this baby had several episodes of cyanosis and persistent tachypnea. After postural measures to help drain fluid and some blow-by O2, the tachypnea persisted. After about one hour, baby A was transferred to the hospital for observation. Chest x-ray showed fluid in the lungs only and he was placed on IV antibiotics as a routine precautionary intervention. His breathing normalized and chest x-ray cleared within 24 hours. Due to hospital policy, he was observed for another 24 hours and was then released.

**Details on maternal seizure transport**

Patient began care with SJF at 27 weeks with IVF di-di twins desiring a home birth option, also receiving concurrent prenatal care with Kaiser Permanente. She developed mild cholestasis of pregnancy and then a falling platelet count to 111,000, minimally elevated AST (normal ALT) and BPs in the 130-140/80 range without proteinuria or edema. She did not want a hospital birth. At 37 6/7 weeks she had a membrane sweep to try to encourage labor with vertex/vertex twins. She had some contractions through the night and drank a castor oil cocktail the following morning. Labor progressed to about 4-5 cm, at which point with her consent AROM of clear fluid was done. FHRs were always in the normal 130-140s range with VSS.

She began assisted pushing with an anterior lip while reducing the cervix. After pushing for about 90 minutes with little progress felt to be mostly due to OP positioning, the option of a vacuum assisted birth was discussed. She readily accepted this option as she was exhausted. Mityvac vacuum was placed without difficulty. There was good rotation and descent to OA position and a quick expulsion of twin A. Good transition with delayed cord clamping and Apgar score of 8 & 9. Weight was 5 pounds 4 ounces.

FHR of baby B was stable in the 130s. Contractions returned within 5-7 minutes and a vaginal exam revealed Twin B now with a foot presenting. FHR remained very stable. Ultrasound showed the baby was now in a back-up transverse lie with a foot and hand presenting. We had previously discussed this possibility during the prenatal visits and the use of a breech extraction. AROM of clear fluid was done. Over a period of 7 minutes and multiple attempts with fundal manipulation, the other foot was brought down from full extension up toward the fetal head. During this time the paramedics were called.

Both feet and legs were then delivered with baby in sacrum anterior position. Both arms were above the fetal head and the right arm was delivered easily with the Lovset maneuver but the left arm was much more difficult. In the process of sweeping it around, the left humerus was broken. The head was then delivered with the MSV maneuver 17 minutes after Twin A. Apgar scores were 3, 7 & 9.  Weight of twin B was 6 pounds 2 ounces. Baby remained with the mother and paramedics were sent away.

Mother’s vital signs and orientation were stable and over the next half hour the babies were on mom’s chest with attempts at latching. After about 35 minutes there was some bleeding and it was noted that the placenta was not detaching. A manual removal of the placenta was then performed. EBL was 1000 cc to this point. Mom was amazingly cooperative and tolerant of all the manipulation over the past hour.

She was given a new IV and a liter of LR with 20 units of Pitocin along with 10 units of Pitocin IM. We also gave her 1 gram of Cefazolin IV. Within the next hour or so, things had seemed to calm down when mom complained of not feeling well and then had about a 45-60 second full tonic-clonic seizure. Paramedics were once again called. Mom was initially post-ictal but was responding by the time the paramedics were moving her onto a gurney. Her O2 saturation was 95% on room air, BP was 120s/70s. Her pulse was 140 and she remained afebrile. She was observed on magnesium sulfate for 36 hours at the hospital and then discharged.

After the maternal transport, the babies remained home with the father and his mother and sister. They had a supply of frozen donor breastmilk available, and the adults were instructed in the technique of finger and syringe feeding.
