# Supplementary material for: Twin home birth: Outcomes of 100 sets of twins in the care of a single practitioner
Source: PLoS One. 2024 Dec 11;19(12):e0313941. doi: 10.1371/journal.pone.0313941 (PMC11633979; doi:10.1371/journal.pone.0313941)
Supplement: Attachment — Submitted filename: Point by Point response Oct 29.docx [file pone.0313941.s007.docx]

6. Review Comments to the Author

**Reviewer #3:**

ABSTRACT: The statement in lines 23-25 should be recasted and grammar corrected.

- We corrected the grammar in these lines.

METHODS: How was the data recorded?

- We added further clarification on page 8, lines 217-218 to note that SJF excerpted the relevant information from the women’s medical records into an Excel spreadsheet.

RESULTS: Under induction vs spontaneous labour, age 283-- how were the women whose pregnancies had passed their expected date of delivery manged?

- As noted in the paragraph under “induction vs. spontaneous labor”, SJF offered a different standard of care and did not mandate delivery by 37 or 38 weeks. Instead, after counseling and informed consent discussions, the normal practice was to await labor. We added another sentence in this section clarifying SJF’s normal practices for clients at or past 40 weeks.

Lines 301-302- for a statement of this nature, figures showing how significance was determined should be included.

- The numbers were too small to calculate p-values; we have decided to omit this sentence to avoid confusion.

DISCUSSION: In lines 316-317, was the 3rd degree laceration which was repaired at home repaired under anaesthesia in the house? If so what type?

- It was repaired with local anesthesia (lidocaine)

In lines 368-369, the statement is not very clear, based on the results of their study which of the birth or indications of the instrumental vaginal deliveries are the authors referring to? Similarly, in lines 370-371, the authors should state for clarity which of the situations they would have managed otherwise and why they did not do so.

- We added some clarification in the paper, noting that SJF’s use of instrumental delivery is related to his experience and skillset. Each case requires an individualized judgment call and is situational (mother’s exhaustion levels, anxiety in the room, etc.). It’s impossible to predict the outcomes of each specific birth with a different care provider who could not use instrumental delivery. We wish to stress that we cannot point to each individual birth and predict the specific outcomes had a midwife been present versus an obstetrician. However, SJF feels that, in general, a midwife may been able to resolve some situations in other ways and may have transported other situations earlier.

The statement in lines 555-556 with quoted figures ideally should be referenced,

- If you are referring to Cynthia Caillagh’s data, it is currently unpublished and RF had personal correspondence with her prior to her death. There is not a formal reference available as her data is not yet published. We added a parenthetical citation to clarify this.
